# Hypomyelinating Leukodystrophy 7 (HLD7)-Associated Mutation of POLR3A Is Related to Defective Oligodendroglial Cell Differentiation, Which Is Ameliorated by Ibuprofen

**DOI:** 10.3390/neurolint14010002

**Published:** 2021-12-22

**Authors:** Sui Sawaguchi, Kenji Tago, Hiroaki Oizumi, Katsuya Ohbuchi, Masahiro Yamamoto, Kazushige Mizoguchi, Yuki Miyamoto, Junji Yamauchi

**Affiliations:** 1 Laboratory of Molecular Neurology, Tokyo University of Pharmacy and Life Sciences, Hachioji, Tokyo 192-0392, Japan; s167023@toyaku.ac.jp (S.S.); miyamoto-y@ncchd.go.jp (Y.M.); 2 Department of Biochemistry, Jichi Medical University, Shimotsuke 321-0498, Japan; ktago@jichi.ac.jp; 3 Tsumura Research Laboratories, Tsumura & Co., Inashiki 200-1192, Japan; ooizumi_hiroaki@mail.tsumura.co.jp (H.O.); oobuchi_katsuya@mail.tsumura.co.jp (K.O.); hirokoma@h.email.ne.jp (M.Y.); mizoguchi_kazushige@mail.tsumura.co.jp (K.M.); 4 Department of Pharmacology, National Research Institute for Child Health and Development, Setagaya, Tokyo 157-8535, Japan

**Keywords:** Pelizaeus-Merzbacher disease (PMD), hypomyelinating leukodystrophy (HLD), POLR3A, lysosome, oligodendrocyte, ibuprofen

## Abstract

Hypomyelinating leukodystrophy 7 (HLD7) is an autosomal recessive oligodendroglial cell-related myelin disease, which is associated with some nucleotide mutations of the RNA polymerase 3 subunit a (polr3a) gene. POLR3A is composed of the catalytic core of RNA polymerase III synthesizing non-coding RNAs, such as rRNA and tRNA. Here, we show that an HLD7-associated nonsense mutation of Arg140-to-Ter (R140X) primarily localizes POLR3A proteins as protein aggregates into lysosomes in mouse oligodendroglial FBD−102b cells, whereas the wild type proteins are not localized in lysosomes. Expression of the R140X mutant proteins, but not the wild type proteins, in cells decreased signaling through the mechanistic target of rapamycin (mTOR), controlling signal transduction around lysosomes. While cells harboring the wild type constructs exhibited phenotypes with widespread membranes with myelin marker protein expression following the induction of differentiation, cells harboring the R140X mutant constructs did not exhibit them. Ibuprofen, a non-steroidal anti-inflammatory drug (NSAID), which is also known as an mTOR signaling activator, ameliorated defects in differentiation with myelin marker protein expression and the related signaling in cells harboring the R140X mutant constructs. Collectively, HLD7-associated POLR3A mutant proteins are localized in lysosomes where they decrease mTOR signaling, inhibiting cell morphological differentiation. Importantly, ibuprofen reverses undifferentiated phenotypes. These findings may reveal some of the pathological mechanisms underlying HLD7 and their amelioration at the molecular and cellular levels.

## 1. Introduction

Hypomyelinating leukodystrophies (HLDs) are a recently classified group of hereditary neuropathies, which are primarily linked to oligodendrocytes (also called oligodendroglial cells) [1,2,3,4]. These diseases are rare, affecting one out of every 250,000 to 500,000 people. HLD1 is also called Pelizaeus-Merzbacher disease (PMD) and is prototypic of all HLDs. The gene responsible for HLD1 encodes proteolipid protein 1 (PLP1) [4]. PLP1 is the major myelin structural protein generated by oligodendroglial cells in the central nervous system (CNS) [5,6,7,8]. Myelin sheaths contribute not only to the propagation of saltatory conduction, but also to protecting neuronal axons from various stresses, such as physical and physiological stresses [5,6,7,8]. Therefore, they play an important role in nerve functions.

Advances in DNA sequencing technologies, such as next-generation sequencing (NGS), have enabled the identification of many expected and unexpected disease-responsible or related genes [3]. The gene responsible for HLD7 (see the OMIM website: https://omim.org/entry/607694 accessed on 1 April 2020; References [9,10]) is one unexpected result of NGS. It encodes the RNA polymerase III subunit A (POLR3A) protein. POLR3A is composed of the catalytic core of RNA polymerase III synthesizing non-coding RNAs, such as 5S rRNA and tRNA (see the gene website: https://www.ncbi.nlm.nih.gov/gene/11128 accessed on 1 April 2020). HLD7 is associated with some of the POLR3A mutations and appears likely to be caused by its loss-of-function [9,10]. Brain imaging demonstrates that HLD7 displays a typical HLD with a thin corpus callosum and other brain regions [9,10]. HLD7 is known to be HLD with hypodontia and hypogonadotropic hypogonadism [9,10]. Similarly, mutations of the gene encoding POLR3B are associated with HLD8 [10,11]. Therefore, HLD7 and HLD8 are also called 4H leukodystrophy (the fourth H referring to hypomyelinating). To date, no therapeutic strategy for HLD7 has been established [12].

HLD7-associated mutation of POLR3A indeed decreases the expression levels of POLR3A proteins. Although some residual mutant proteins are present in pathological cells and tissues [9,10,13], it remains unclear how these mutant proteins cause the molecular and cellular pathological effects of HLD7. Here, we describe how, similar to other HLD-related cell phenotypes, HLD7-associated nonsense mutation of Arg140-to-Ter (R140X) localizes POLR3A proteins as aggregates into lysosomes in the mouse cell line FBD−102b as an oligodendroglial cell model. Consistently, expression of the R140X mutant proteins in cells decreased lysosome-related signals, including the mechanistic target of rapamycin (mTOR) signaling, such as ribosomal S6 and translational 4E-BP1 protein phosphorylation. However, the wild type proteins were not mainly localized in lysosomes and retained their phosphorylation. Cells harboring the wild type constructs exhibited differentiated phenotypes. In contrast, cells harboring the R140X mutant constructs blunted morphological differentiation. In addition, the phenotypes of cells harboring the R140X mutant constructs were reversed by ibuprofen [14,15,16,17], which is a non-steroidal anti-inflammatory drug (NSAID) and mTOR activator. These results suggest a possible pathological mechanistic basis of HLD7 and a possible method of treating it. In particular, the HLD7-associated mutation of POLR3A proteins affects oligodendroglial cell morphological differentiation through lysosome-related mTOR signaling, possibly presenting some molecular and cellular pathological mechanisms underlying HLD7.

## 2. Material and Methods

### 2.1. Primary and Secondary Antibodies, Inhibitors, and Plasmid Constructions

Antibodies and inhibitors are listed in Table 1. The full-length human polr3a gene (Gene ID: 11128), which is inserted into a Halo-tag-expressing pFN21A (Kazusa Genome Technologies, Chiba, Japan), was purchased from Kazusa Genome Technologies and Promega (Madison, WI, USA). The polr3a cDNA harboring the Arg140-to-Ter (R140X) mutation (OMIM ID 614258) was generated with a Gflex DNA polymerase (Takara Bio, Shiga, Japan)-based method using the full-length human polr3a gene as template. DNA sequences were confirmed by the Fasmac sequencing service (Kanagawa, Japan).

### 2.2. Cell Culture and Differentiation

Cells from the oligodendroglial FBD−102b cell line (a mouse brain oligodendroglial precursor cell line) were kindly provided by Dr. Y. Tomo-oka (Tokyo University of Science, Chiba, Japan; and Riken, Saitama, Japan). FBD−102b cells [18,19,20,21,22] were cultured on standard cell culture dishes (Greiner, Oberösterreich, Germany) in a culture medium consisting of DMEM/Nutrient Mixture F-12 (Nacalai Tesque, Kyoto, Japan) containing 10% heat-inactivated FBS (Thermo Fisher Scientific, Waltham, MA, USA) and PenStrep (Thermo Fisher Scientific) in 5% CO_2_ at 37 °C. To induce differentiation, FBD−102b cells were cultured for 5 days in the same culture medium without FBS on cell culture dishes with advanced TC polymer surface modification (Greiner) in 5% CO_2_ at 37 °C [18,19,20,21,22]. We incubated cells with or without 300 μM ibuprofen [14,15,16,17] and/or 500 nM AH6809 (IC_50_ = 6.8 nM for prostaglandin EP1 receptor in a cell-free system; see the Tocris Biological Activity website, https://www.tocris.com/ accessed on 1 April 2020) for 2 days. In addition, we changed the culture medium into a newly-prepared medium containing ibuprofen and/or AH6809, and further incubated cells with each or both of them for 3 days. Cells with widespread membranes (more than 75 μm square fields; Image J software [Bethesda, MD, USA]) were identified as differentiated [18,19,20,21,22]. We confirmed that FBD−102b cells were viable under each experimental condition by verifying that the attached trypan-blue (Nacalai Tesque)-incorporating cells made up less than 5% of all the cells in each culture. Along with these observations, the expression levels of myelin marker proteins were confirmed by immunoblotting techniques. FBD−102b cells form widespread membranes and express myelin marker proteins [18,19,20,21,22]. However, it is unlikely that FBD−102b cells are able to achieve the terminal differentiated phenotype, since they fail to exhibit the myelin web-like membrane structures needed to wrap neuronal axons with their membranes.

### 2.3. Transfection into Cells

Cells were transfected with the respective plasmids using a ScreenFect A or ScreenFect A Plus transfection kit (Fujifilm, Tokyo, Japan) according to the manufacturer’s instructions. The medium was replaced 4 h after transfection. Transfected cells were generally used for experiments 48 h after transfection in biochemical experiments. We confirmed that FBD−102b cells were viable under each experimental condition by verifying that the attached trypan-blue (Nacalai Tesque)-incorporating cells made up less than 5% of all the cells in each culture.

### 2.4. Confocal Microscopic Images

Coverslips loaded with cells fixed with 4% paraformaldehyde or 100% cold methanol were blocked with Blocking One reagent (Nacalai Tesque). Then, these were incubated with primary antibodies followed by secondary antibodies conjugated with Alexa Fluor dyes and/or Halo-tag ligand reagent (Promega), according to the manufacturer’s instructions. The coverslips on each slide glass were mounted with Vectashield reagent (Vector Laboratories, Burlingame, CA, USA). TIFF images were collected through a microscope equipped with a laser-scanning Fluoview apparatus (FV1000D or FV1200, Olympus, Tokyo, Japan) and processed using Fluoview software (Olympus). The resulting color images were analyzed in Image J software. Each image in each figure is representative of three independent experimental results.

### 2.5. Polyacrylamide Gel Electrophoresis and Immunoblotting

Cells were lysed in lysis buffer A (50 mM HEPES-NaOH, pH 7.5, 150 mM NaCl, 20 mM MgCl_2_, 1 mM phenylmethane sulfonylfluoride, 1 μg/mL leupeptin, 1 mM EDTA, 1 mM Na_3_VO_4_, 10 mM NaF, and 0.5% NP-40) [23,24]. After centrifugation, for non-denatured and denatured conditions, the supernatants were incubated with a non-denaturing sample buffer (also called the native polyacrylamide gel sample buffer; Nacalai Tesque) and the denaturing sample buffer (Nacalai Tesque), respectively. Then, the samples were separated on non-denaturing or denaturing polyacrylamide gels (Nacalai Tesque). The electrophoretically separated proteins were transferred onto polyvinylidene difluoride membranes (Merck-Millipore, Darmstadt, Germany) and blocked with Blocking One reagent, then immunoblotted with primary antibodies followed by secondary antibodies conjugated with HRP proteins. The bound antibodies were detected by means of X-ray film exposure using ImmunoStar Zeta reagent (Fujifilm). Images were captured as TIFF files using Canon LiDE scanners (Canon, Tokyo, Japan) and processed using the accompanying driver software (Canon). The band pixels were measured in Image J software. Each image in each figure is representative of three independent experimental results.

### 2.6. Statistical Analysis

Values are means ± standard deviation (SD) from separate experiments. Intergroup comparisons were made using the unpaired Student’s *t*-test using Excel (Microsoft, Redmond, WA, USA). A one-way analysis of variance (ANOVA) was followed by a Fisher’s protected least significant difference (PLSD) test as a post hoc comparison using StatPlus (AnalystSoft, Walnut, CA, USA). Differences were considered statistically significant when *p* < 0.05.

### 2.7. Ethics Statement

Gene recombination techniques were performed in accordance with a protocol approved by the Tokyo University of Pharmacy and Life Sciences Gene and Animal Care Committee (Approval Nos. LS28-20 and LSR3-011).

## 3. Results

### 3.1. The R140X Mutant Proteins of POLR3A Are Aggregated and Present in Lysosomes in FBD−102b Cells

**Figure 1:**
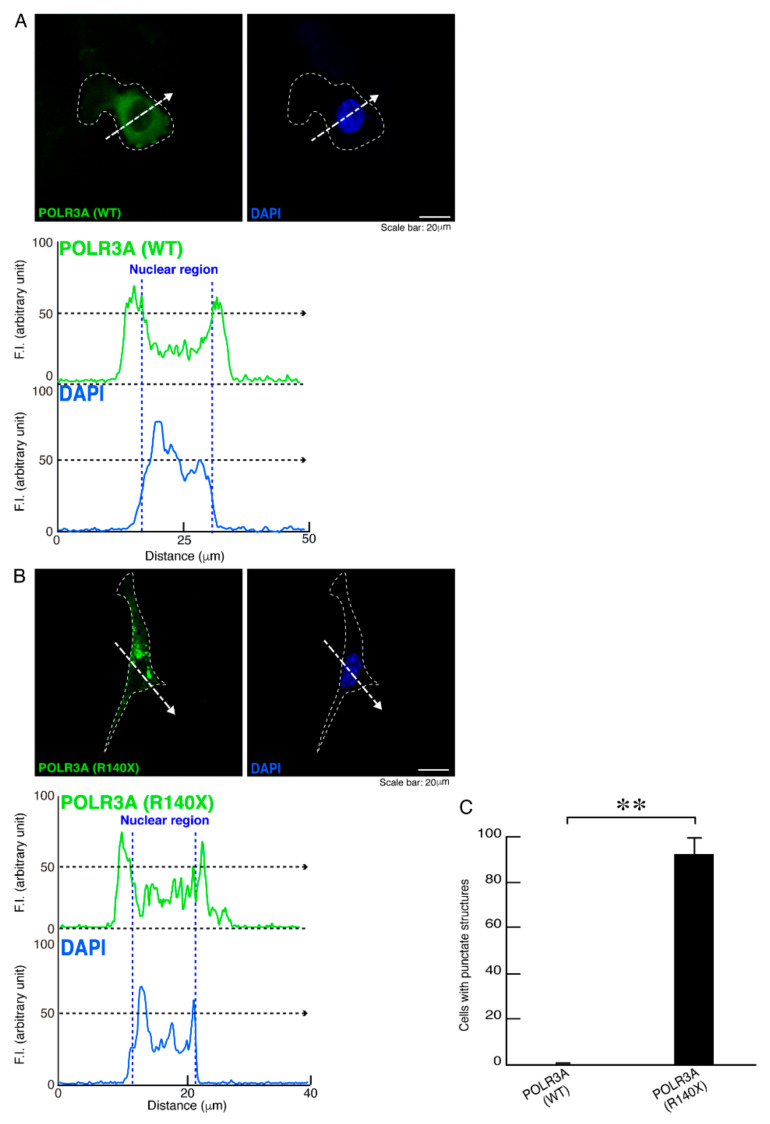
The R140X mutant proteins, but not the wild type proteins, of POLR3A are accumulated in punctate structures in FBD−102b cells. (**A**,**B**) Oligodendroglial FBD−102b cells, surrounded by white lines, were transfected with the plasmid encoding wild type (WT) POLR3A or the R140X mutant constructs. Transfected cells were detected with transfected proteins (green) and nuclear DAPI (blue). Scan plots were performed along the white dotted lines in the direction of the arrows in images. Graphs showing the fluorescence intensities (arbitrary units) along the white dotted lines in the direction of the arrows (black dotted lines in right bottom panels) can be seen in the bottom panels. (**C**) Percentages of cells with punctate structures were statistically assessed (** *p* < 0.01; *n* = 3 fields).

**Figure 2:**
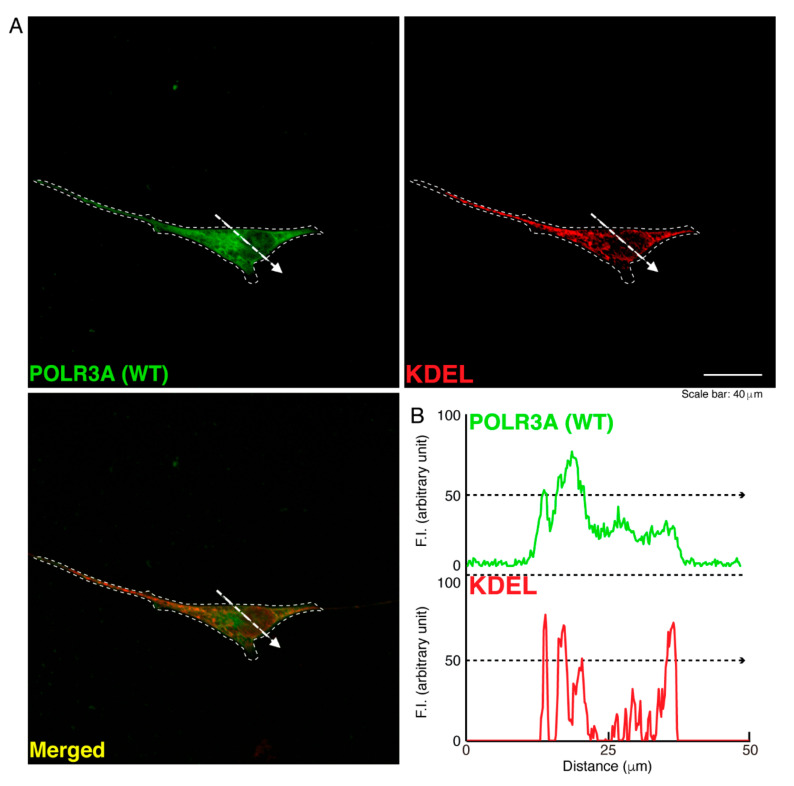
The wild type POLR3A proteins are not co-localized with the ER in cells. (**A**,**B**) FBD−102b cells were transfected with the plasmid encoding the wild type (WT) POLR3A and stained with transfected proteins (green) and an antibody against the KDEL antigen (red). Scan plots were performed along the white dotted lines in the direction of the arrows in the color images (green and red as well as merged images). Graphs showing the fluorescence intensities (arbitrary units) along the white dotted lines in the direction of the arrows can be seen in the right bottom panels (black dotted lines).

**Figure 3:**
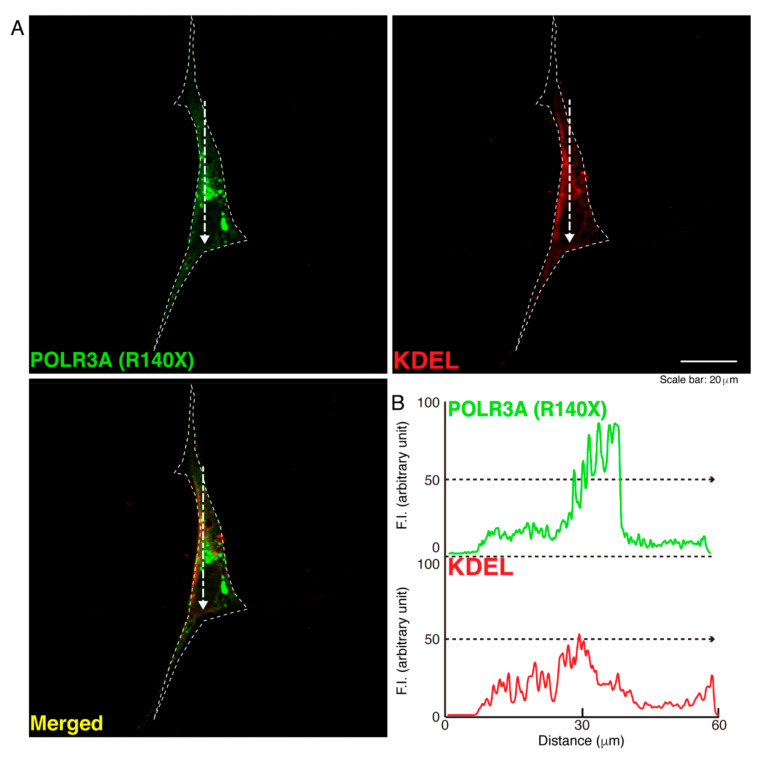
The POLR3A mutant proteins are not co-localized with the ER in cells. (**A**,**B**) FBD−102b cells were transfected with the plasmid encoding the POLR3A R140X construct (green) and stained using an antibody against the KDEL antigen (red). Scan plots were performed along the white dotted lines in the direction of the arrows in the color images (green and red as well as merged images). Graphs showing the fluorescence intensities (arbitrary units) along the white dotted lines in the direction of the arrows can be seen in the right bottom panels (black dotted lines).

**Figure 4:**
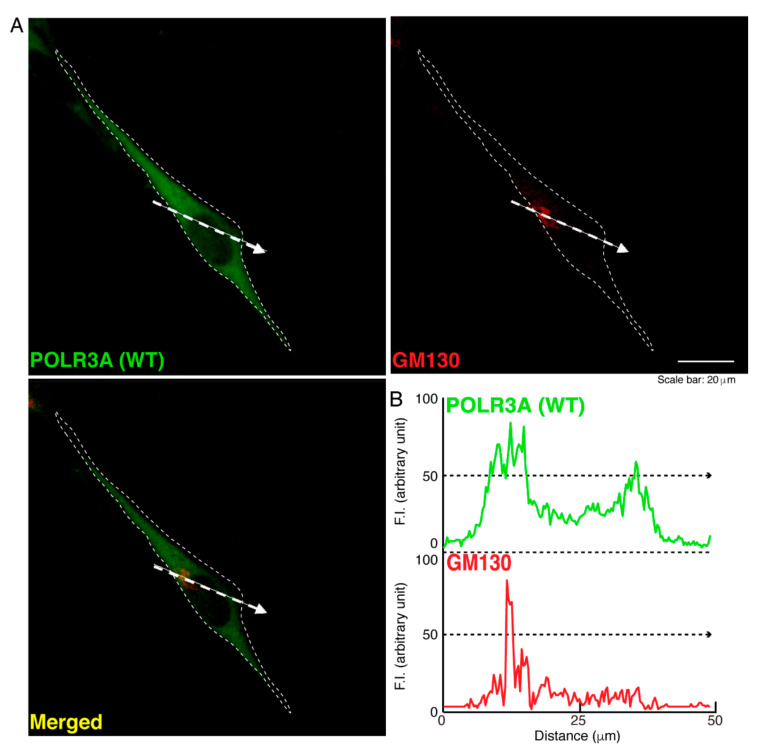
The wild type POLR3A proteins are not co-localized with the Golgi body in cells. (**A**,**B**) FBD−102b cells were transfected with the plasmid encoding the wild type (WT) POLR3A (green) and stained using an antibody against the GM130 antigen (red). Scan plots were performed along the white dotted lines in the direction of the arrows in the color images (green and red as well as merged images). Graphs showing the fluorescence intensities (arbitrary units) along the white dotted lines in the direction of the arrows can be seen in the right bottom panels (black dotted lines).

**Figure 5:**
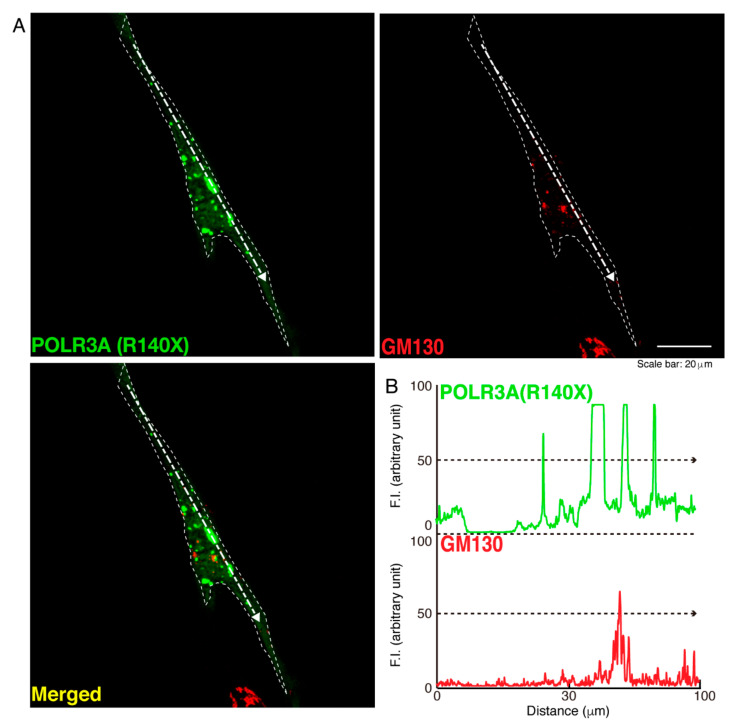
The POLR3A mutant proteins are not co-localized with the Golgi body in cells. (**A**,**B**) FBD−102b cells were transfected with the plasmid encoding the POLR3A R140X construct (green) and stained using an antibody against the GM130 antigen (red). Scan plots were performed along the white dotted lines in the direction of the arrows in the color images (green and red as well as merged images). Graphs showing the fluorescence intensities (arbitrary units) along the white dotted lines in the direction of the arrows can be seen in the right bottom panels (black dotted lines).

**Figure 6:**
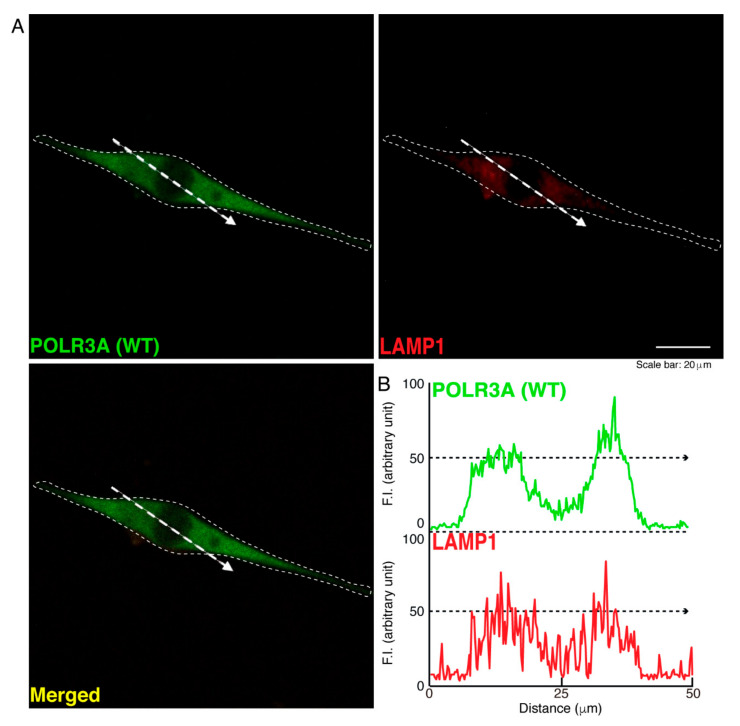
The wild type POLR3A proteins are not co-localized with the lysosome in cells. (**A**,**B**) FBD−102b cells were transfected with the plasmid encoding the wild type (WT) POLR3A (green) and stained using an antibody against the LAMP1 antigen (red). Scan plots were performed along the white dotted lines in the direction of the arrows in the color images (green and red as well as merged images). Graphs showing the fluorescence intensities (arbitrary units) along the white dotted lines in the direction of the arrows can be seen in the right bottom panels (black dotted lines).

**Figure 7:**
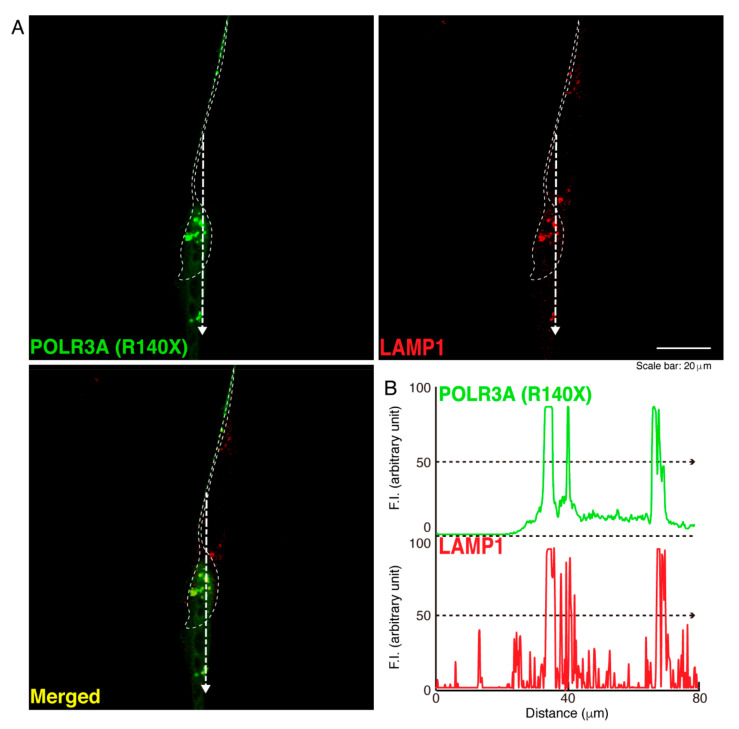
The POLR3A mutant proteins are co-localized with the lysosome in cells. (**A**,**B**) FBD−102b cells were transfected with the plasmid encoding the POLR3A R140X construct (green) and stained using an antibody against the LAMP1 antigen (red). Scan plots were performed along the white dotted lines in the direction of the arrows in the color images (green and red as well as merged images). Graphs showing the fluorescence intensities (arbitrary units) along the white dotted lines in the direction of the arrows can be seen in the right bottom panels (black dotted lines).

To determine whether, similar to other HLD-related cellular phenotypes [18,19,20,21,22], the R140X mutant proteins of POLR3A form protein aggregates in cells, we transfected the plasmid encoding POLR3A harboring the R140X mutation or the wild type into FBD−102b cells. The transfected cell images showed that the wild type POLR3A proteins were distributed throughout the cytoplasm and slightly in nuclei (Figure 1A). In contrast, the R140X mutant proteins were present in aggregate-like punctate structures by over 90% (Figure 1B,C). Then, to explore which organelle contains punctate structures, we transfected the plasmid encoding constructs harboring the R140X mutation or the wild type into cells and stained these cells with one of the following specific antigens: Lys-Asp-Asn-Leu (KDEL) against the endoplasmic reticulum (ER); Golgi matrix protein of 130 kDa (GM130) against the Golgi body or lysosomal-associated membrane protein 1 (LAMP1) against the lysosome. The wild type and mutant proteins were not co-localized with KDEL antigens (Figure 2A,B and Figure 3A,B). Similar results were obtained in the cases of GM130 antigens (Figure 4A,B and Figure 5A,B). In contrast, the wild type proteins were not co-localized with LAMP1 antigens, whereas mutant proteins were co-localized with LAMP1 antigens (Figure 6A,B and Figure 7A,B), indicating that the R140X mutant proteins, but not the wild type proteins, of POLR3A are primarily localized in lysosomes. Furthermore, we examined if the R140X mutant proteins are localized in autophagosomes to induce autophagy. We stained cells with an antibody against LC3, which is an autophagosome marker. The mutant proteins, but not the wild type proteins, were contained in autophagosomes (Figures S1 and S2).

**Figure 8:**
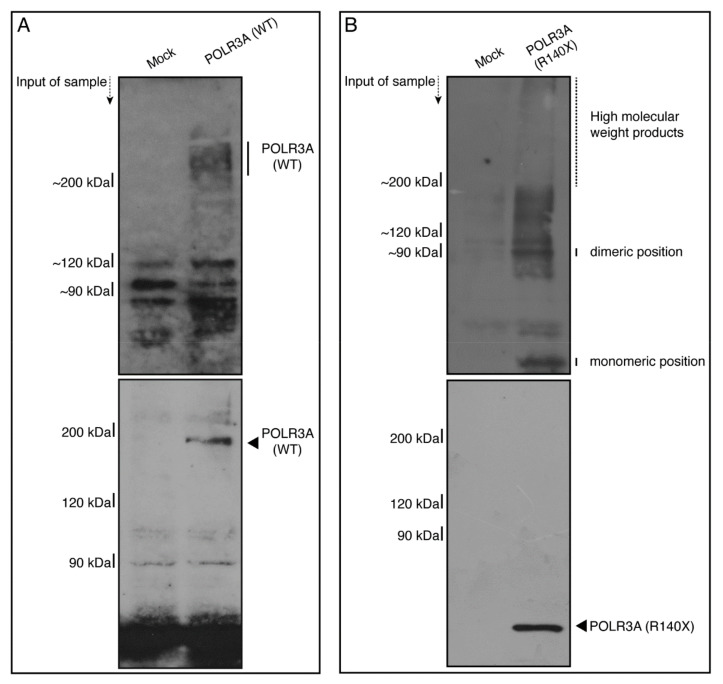
The POLR3A mutant proteins, but not the wild type proteins, exhibit dimeric and high-molecular-weight structures in non-denaturing polyacrylamide gel electrophoresis. (**A**,**B**) The lysates of FBD−102b cells transfected with an empty vector or with a plasmid encoding the wild type (WT) or POLR3A R140X construct were subjected to non-denaturing (upper images) and denaturing (lower images) polyacrylamide gel electrophoresis and detected using immunoblotting. The positions corresponding to the molecular weight of the wild type POLR3A monomer or the POLR3A R140X monomeric or polymeric (including high-molecular-weight products) structures are shown.

Moreover, we sought to determine whether in non-denatured polyacrylamide gel electrophoresis, the R140X mutant proteins display protein bands with a high molecular weight, which is a hallmark of aggregation [4,18,19,20,21,22]. In denatured polyacrylamide gel electrophoresis, the wild type and mutant proteins corresponded to the respective molecular weights. In contrast, mutant proteins, but not wild type proteins, exhibited immunoreactive bands with dimeric and high molecular weight positions (Figure 8A,B; see Figure S3 for original size gel scan images). This suggests that the R140X mutant proteins are present as protein aggregates.

### 3.2. Morphological Differentiation Is Inhibited in Cells Harboring the R140X Mutant Constructs of POLR3A

**Figure 9:**
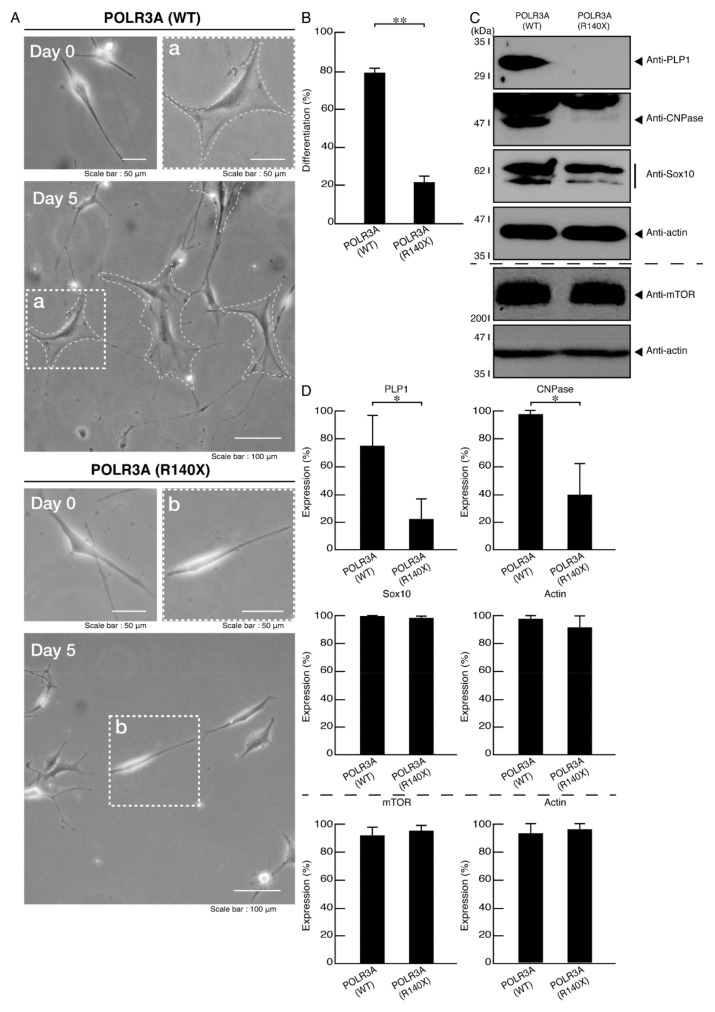
Cells harboring the POLR3A mutants, but not the wild types, fail to exhibit morphological differentiation. (**A**) FBD−102b cells harboring the wild type (WT) or R140X construct were allowed to differentiate for 5 days. Some cells in the bottom images are surrounded by white dotted lines (a and b). The square fields a and b indicated by dotted lines in the bottom panels are magnified in the upper panels a and b. Images of cells at day 0 are also shown. (**B**) Differentiated cells were statistically assessed (** *p* < 0.01; *n* = 3 fields). (**C**) The lysates of the respective cells were immunoblotted with an antibody against the differentiation (myelin) marker proteins PLP1 and CNPase, oligodendrocyte lineage cell marker Sox10, mTOR, and control actin. (**D**) Their expression levels are shown statistically compared to their respective controls (* *p* < 0.05; *n* = 3 blots).

FBD−102b cells display oligodendroglial cell differentiated phenotypes with widespread membranes at 3 to 5 days following the induction of differentiation [18,19,20,21,22]. We checked the possibility that the R140X mutation of POLR3A leads to the inhibition of differentiation. Following the induction of differentiation, cells harboring the wild type constructs exhibited differentiated phenotypes. On the other hand, cells harboring the R140X mutant constructs did not exhibit them (Figure 9A,B), with decreased expression of differentiation (and myelin) marker proteins PLP1 and CNPase in cells (Figure 9C,D). Oligodendrocyte lineage cell marker Sox10 and control actin protein levels were comparable in both cells. In addition, the expression levels of mTOR proteins were comparable in both cells. It is unlikely that the mutation of POLR3A affects the expression levels of mTOR proteins.

**Figure 10:**
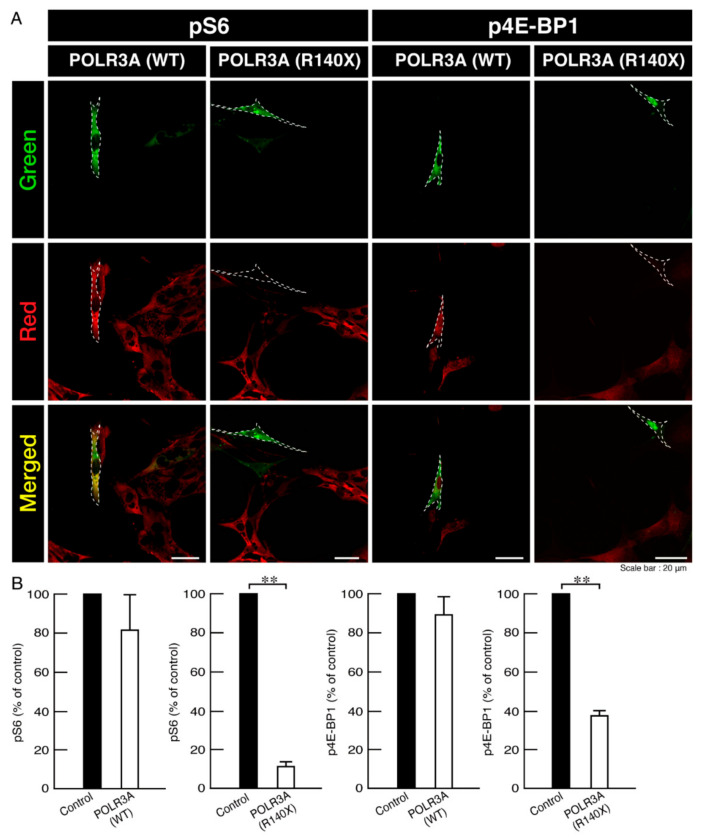
Cells harboring the POLR3A mutants, but not the wild types, decrease phosphorylation levels of ribosomal S6 and 4E-BP1 proteins. (**A**,**B**) FBD−102b cells harboring the wild type (WT) or R140X construct (green) were stained with an anti-(pS240 and pS244) ribosomal S6 protein (pS6) or anti-(pT37)4E-BP1 (p4E-BP1) antibody (red) whose phosphorylation acts downstream of mTOR signaling. Their phosphorylation levels are shown statistically compared to those in their respective control cells under each image (** *p* < 0.01; *n* = 3 fields).

Signaling around lysosomes is critically associated with mTOR [25,26], which is also important for oligodendroglial cell differentiation and myelination [27,28,29,30]. Therefore, we examined whether the expression of the R140X mutant proteins in cells is related to the phosphorylation of ribosomal S6 and 4E-BP1 proteins, which performs the downstream phosphorylation of mTOR signaling [25,26]. The phosphorylation plays roles in protein translation and mRNA transcription [25,26]. Cells expressing the wild type POLR3A proteins retained the active, phosphorylated states of ribosomal S6 proteins as well as 4E-BP1 ones. In contrast, cells expressing mutant proteins showed decreased phosphorylated states (Figure 10A,B), suggesting that the R140X mutation is related to decreased oligodendroglial morphological differentiation with decreased mTOR signaling (such as ribosomal S6 and 4E-BP1 signaling).

### 3.3. Ibuprofen Reverses Phenotypes in Cells Harboring the R140X Mutant Constructs of POLR3A

**Figure 11:**
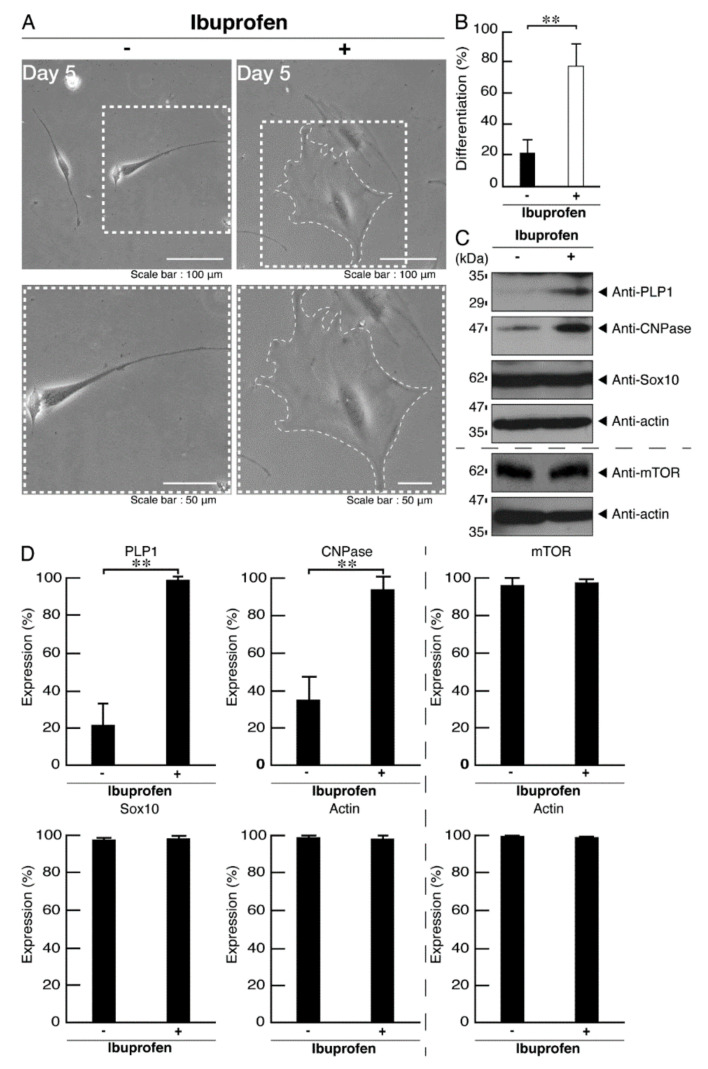
Ibuprofen reverses phenotypes in cells harboring the POLR3A mutants. (**A**) FBD−102b cells harboring the R140X constructs were allowed to differentiate in the presence or absence of ibuprofen for 5 days. Some cells in the upper images are surrounded by white dotted lines. The square fields indicated by dotted lines in the upper panels are magnified in the respective lower panels. (**B**) Differentiated cells were statistically assessed (** *p* < 0.01; *n* = 3 fields). (**C**) The lysates of the respective cells were immunoblotted with an antibody against PLP1, CNPase, Sox10, mTOR, and control actin. (**D**) Their expression levels are shown statistically compared to their respective controls (** *p* < 0.01; *n* = 3 blots).

**Figure 12:**
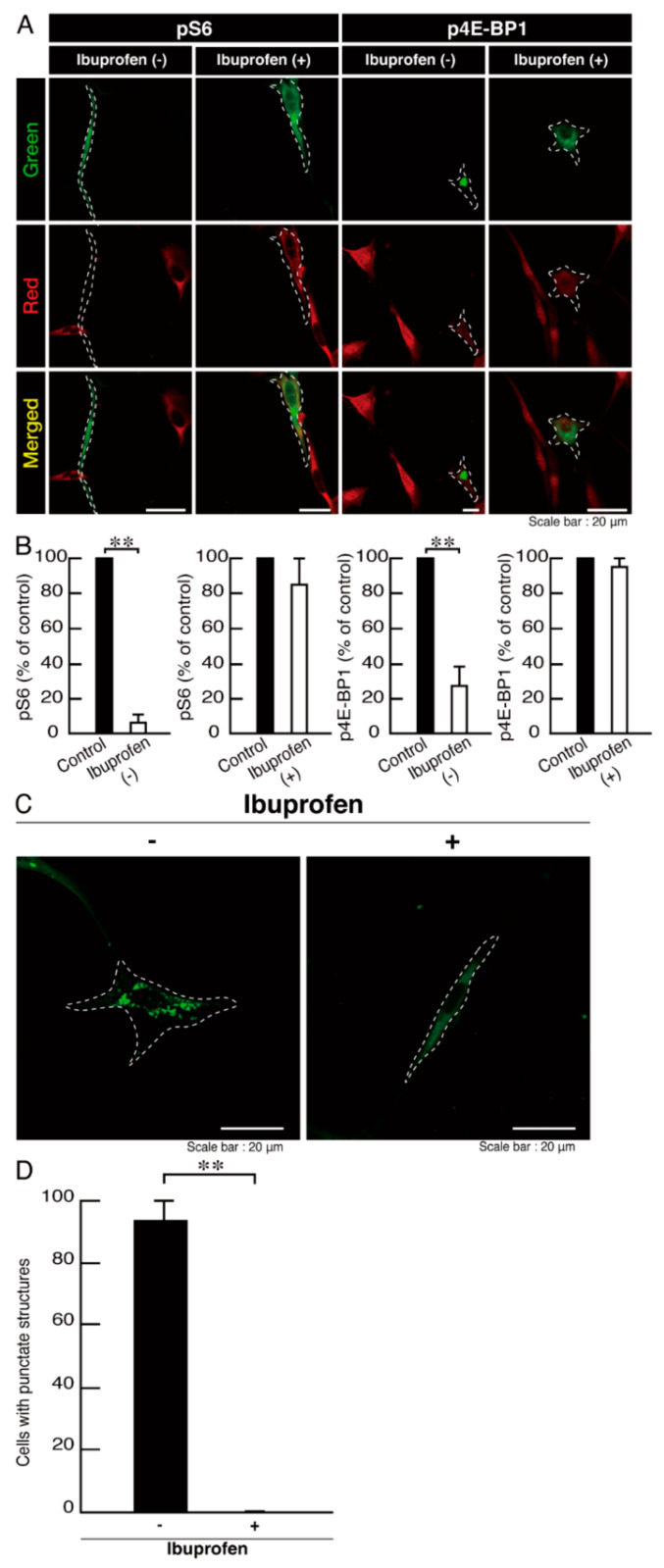
Ibuprofen reverses phosphorylation levels of ribosomal S6 and 4E-BP1 proteins and aggregate-like punctate structures in cells harboring the POLR3A mutants. (**A**,**B**) FBD−102b cells harboring the R140X constructs (green) in the presence or absence of ibuprofen were stained with an anti-(pS240 and pS244) ribosomal S6 protein (pS6) or anti-(pT37)4E-BP1 (p4E-BP1) antibody (red). Their phosphorylation levels are shown statistically compared to those in their respective control cells under each image (** *p* < 0.01; *n* = 3 fields). (**C**,**D**) Cells were treated with or without ibuprofen. Percentages of cells with punctate structures were statistically assessed (** *p* < 0.01; *n* = 3 fields).

Ibuprofen is a NSAID that is known to be an activator of mTOR signaling [14,15,16,17]. It has also been reported to have broad neuroprotective effects [15,17]. Therefore, we attempted to determine whether ibuprofen can reverse undifferentiated phenotypes mediated by the R140X mutation of POLR3A. The treatment with ibuprofen resulted in differentiated phenotypes in cells harboring the R140X mutant constructs (Figure 11A,B). The results of changes in these cellular phenotypes were supported by increased expression of PLP1 and CNPase (Figure 11C,D). Expression levels of Sox10 and actin were comparable in the presence or absence of ibuprofen. In addition, expression levels of mTOR proteins are comparable in the presence or absence of ibuprofen. It is unlikely that ibuprofen affects the expression levels of mTOR proteins. In cells harboring the R140X mutant constructs, ibuprofen reversed the phosphorylation levels of ribosomal S6 and 4E-BP1 proteins (Figure 12A,B) and aggregation-like punctate structures (Figure 12C,D).

**Figure 13:**
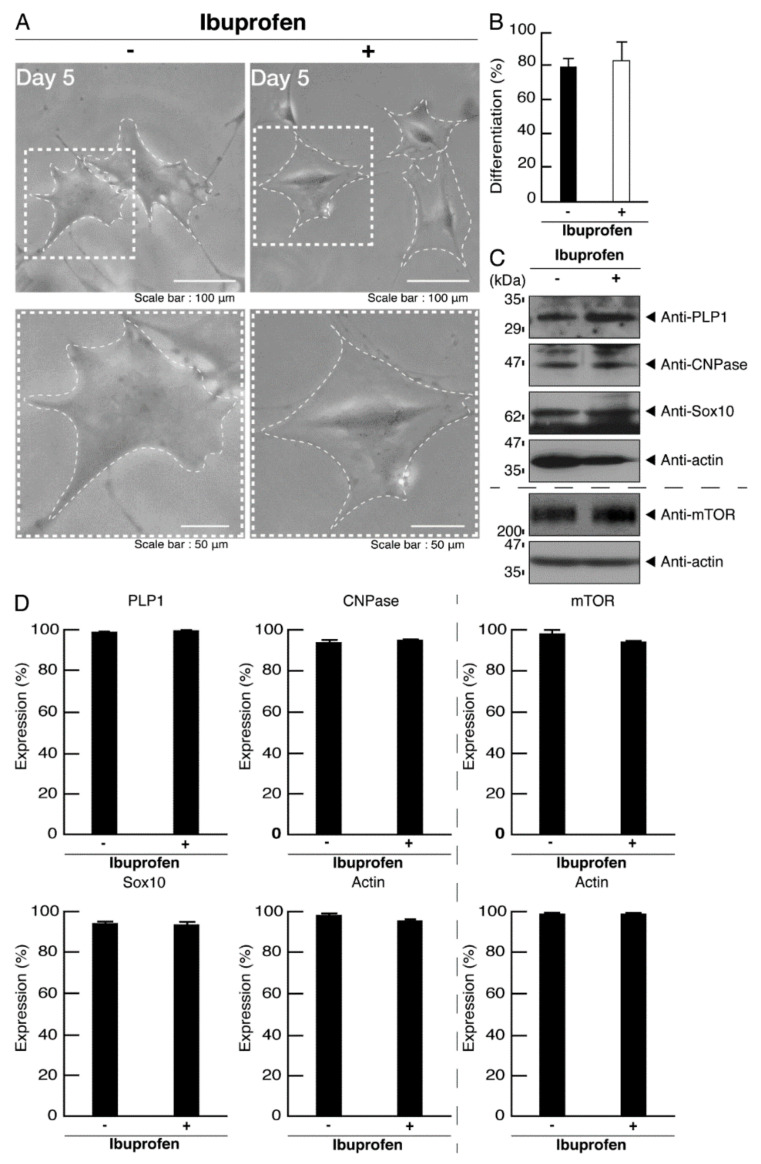
Ibuprofen does not have significant effects on phenotypes in cells harboring the wild type POLR3A. (**A**) FBD−102b cells harboring the wild type POLR3A were allowed to differentiate in the presence or absence of ibuprofen for 5 days. Some cells in the upper images are surrounded by white dotted lines. The square fields indicated by dotted lines in the upper panels are magnified in the respective lower panels. (**B**) Differentiated cells were statistically assessed (*n* = 3 fields). (**C**) The lysates of the respective cells were immunoblotted with an antibody against PLP1, CNPase, Sox10, mTOR, and control actin. (**D**) Their expression levels are shown statistically compared to their respective controls (*n* = 3 blots).

**Figure 14:**
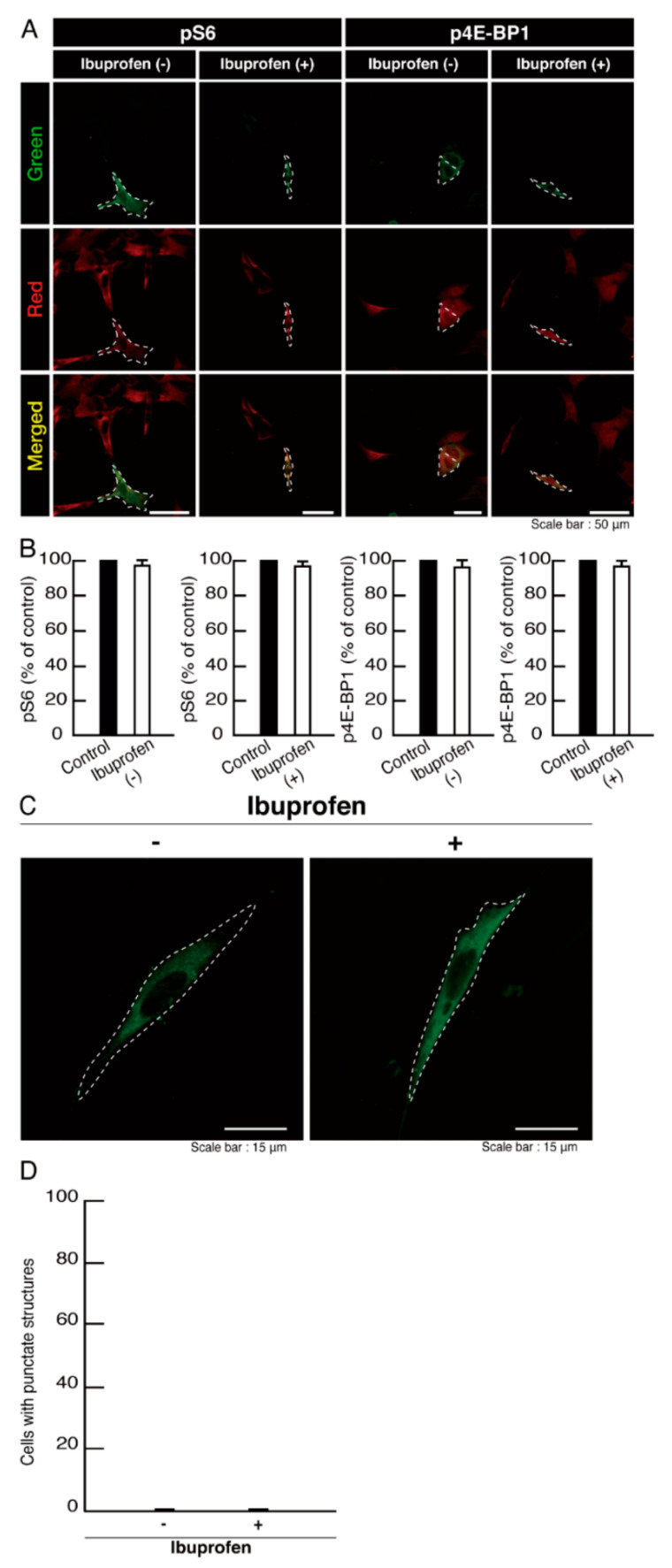
Ibuprofen does not have significant effects on phosphorylation levels of ribosomal S6 and 4E-BP1 proteins and aggregate-like punctate structures in cells harboring the wild type POLR3A. (**A**,**B**) FBD−102b cells harboring the wild type (green) in the presence or absence of ibuprofen were stained with an anti-(pS240 and pS244) ribosomal S6 protein (pS6) or anti-(pT37)4E-BP1 (p4E-BP1) antibody (red). Their phosphorylation levels are shown statistically compared to those in their respective control cells under each image (*n* = 3 fields). (**C**,**D**) Cells were treated with or without ibuprofen. Percentages of cells with punctate structures were statistically assessed (*n* = 3 fields).

In contrast, ibuprofen did not exhibit significant effects on the morphological differentiation of cells harboring the wild type constructs (Figure 13A,B). Similar results were obtained in the case of differentiation marker proteins and mTOR proteins (Figure 13C,D), phosphorylation levels of ribosomal S6 and 4E-BP1 proteins (Figure 14A,B), and the intracellular distribution of POLR3A proteins (Figure 14C,D). Ibuprofen appears likely to reverse phenotypes in cells harboring the R140X mutant constructs of POLR3A. It is unlikely that ibuprofen affects the expression levels of mTOR proteins.

**Figure 15:**
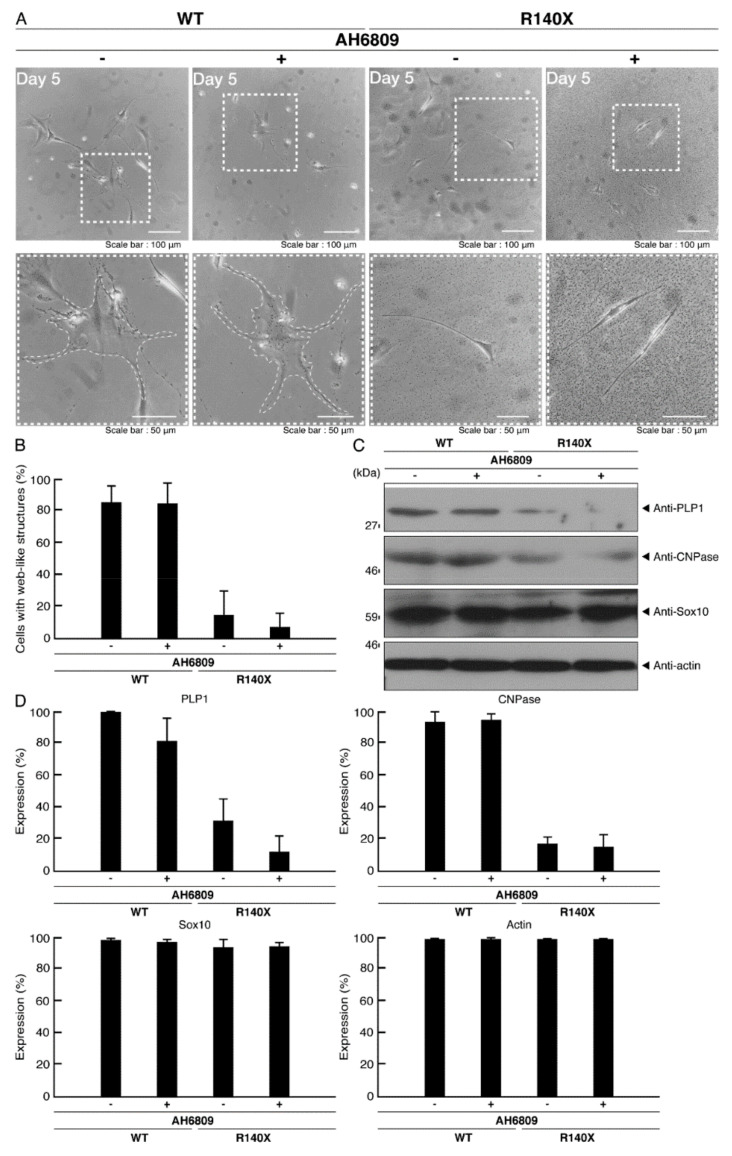
AH6809 does not have significant effects on morphological differentiation. (**A**) FBD−102b cells harboring the wild type (WT) or R140X POLR3A were allowed to differentiate in the presence or absence of AH6809 for 5 days. Some cells in upper images are surrounded by white dotted lines. The square fields indicated by dotted lines in the upper panels are magnified in the respective lower panels. (**B**) Differentiated cells were statistically assessed (*n* = 3 fields). (**C**) The lysates of the respective cells were immunoblotted with an antibody against differentiation (myelin) marker proteins PLP1 and CNPase, oligodendrocyte lineage cell marker Sox10, and control actin. (**D**) Their expression levels are shown statistically compared to their respective controls (*n* = 3 blots).

Ibuprofen has many effects on cells. It is well established that ibuprofen suppresses the production of prostaglandins and that it exhibits antipyretic and analgesic effects by inhibiting cyclooxygenases, similarly to other NSAIDs [14]. In order to examine whether inhibition of cyclooxygenases is associated with the effect of ibuprofen on morphological differentiation, we treated AH6809 with cells. AH6809 primarily acts as a prostaglandin D2 receptor and prostaglandin E2 receptor antagonist with nearly equal affinity. The treatment with AH6809 did not show any effects on morphological differentiation in cells expressing the wild type POLR3A or in ones expressing the R140X mutant (Figure 15A,B). Similar effects were obtained for the expression levels of differentiation marker proteins (Figure 15C,D). It is unlikely that the effects of ibuprofen on morphological differentiation are due to the inhibition of cyclooxygenases.

## 4. Discussion

Brain imaging shows that HLD7 leads to thin myelin sheaths in the corpus callosum and other brain regions [9,10]. Consistent with the in vivo data, we demonstrate that HLD7-associated R140X mutant proteins of POLR3A, but not the wild type proteins, are primarily localized as aggregates in lysosomes in FBD−102b cells, inhibiting the morphological differentiation in FBD−102b cells. These findings are supported by the finding that cells harboring the R140X mutant constructs, but not the wild type, blunted morphological differentiation. In addition, the R140X mutant proteins exhibited the immunoreactive bands at a high molecular weight in non-denaturing polyacrylamide gel electrophoresis. Their aggregated proteins were mainly observed in lysosomes. R140X mutant expressing cells tended to exhibit collective lysosome and ER organelle shapes. Additionally, they exhibited fragmentational Golgi bodies. It is thought that these intracellular organelle phenotypes are one of the pathological forms. Abnormal distributions of these organelles may affect signal transductions around the organelles and disarrange them. Furthermore, these abnormal distributions may also affect the normal functions of organelles, probably blunting cellular differentiation. The fact that the R140X mutation is associated with blunted morphological differentiation might be related to the thin myelin sheaths found in HLD7 patients. Furthermore, the expression of the R140X mutant proteins, but not the wild type proteins, in cells decreased lysosome-related signals including signaling through mTOR, such as phosphorylation of ribosomal S6 proteins and 4E-BP1 proteins. Signaling through mTOR has been known to trigger differentiation and myelin sheath formation in the corpus callosum in mice [27,28]. Therefore, the decreased activities of molecules involved in mTOR signaling might also be associated with the thin myelin sheaths observed in HLD7 patients. However, it remains to be clarified whether brain tissue lysate in HLD7 patients contains lower activities of molecules belonging to mTOR signaling.

The R140X mutant proteins generate shorter proteins with the immature translation stop signal. Therefore, the entire protein structures of POLR3A proteins may be destroyed in order to be incorporated as the aggregate-like structures into intracellular organelles, such as the lysosome. Alternatively, the mutant proteins may generate a new organelle localization signal with a protruding amino acid end for intracellular organelles, such as the lysosome, although precise lysosomal localization signals and structures remain unknown. It is likely that abnormal protein localization in intracellular organelles, such as the lysosome is associated with a decrease in S6 and 4E-BP1 phosphorylation, which acts downstream of mTOR signaling around the lysosome. Moreover, it has been well known that S6 and 4E-BP1 phosphorylation is involved in the regulation of oligodendroglial cell differentiation [27,28]. Taking these things into consideration, it is conceivable that an abnormal protein localization-mediated decrease in S6 and 4E-BP1 phosphorylation is linked to inhibitory differentiation.

No therapeutic strategy has been established for any of the HLDs, including PMD. Several therapeutic, as opposed to merely symptomatic treatments have been attempted in disease model mice. Previous studies in PMD model mice and PMD model mice tissues, have shown that some therapeutic treatments are effective or potentially effective in ameliorating hypomyelination and/or demyelination [31,32,33,34,35,36]. Lonaprisan (also called BAY 86-5044), which is a potent synthetic selective antagonist of the progesterone receptor and a potential treatment of breast cancer, has been shown to increase myelinated axon numbers and to improve poor motor phenotypes [31]. Curcumin, which is a lipophilic polyphenol that has potential uses as an anti-inflammatory, antitumor or antibiotic drug, has also been shown to increase in the number of myelinated axons and to reverse poor motor phenotypes [32]. Interestingly, the ketogenic diet restores oligodendrocyte integrity and increases myelination in the brain [33]. In addition, it has been reported that transplantation of human neural stem cells, but not multipotent human or mouse ES cells or iPS cells, into the brain induces neural regeneration [34,35]. Moreover, deferiprone, which is an iron-chelating chemical used to treat iron overload in thalassemia, reduces oligodendrocyte apoptosis, resulting in upregulated differentiation and, in turn, axonal myelination in oligodendrocytes [36]. It is thought that iron-chelating is effective for PMD patient-derived oligodendrocytes, since they display critical hallmarks of ferroptosis, including abnormal iron metabolism, hypersensitivity to free iron, and lipid peroxidation. While the treatment with lonaprisan, curcumin or a ketogenic diet enters the category of symptomatic treatment, transplantation with human neural stem cells or treatment with deferiprone may be specific for PMD patients. Here, we report that downregulation of mTOR signaling is caused by HLD7-associated mutant proteins in cells. Therefore, if downregulated mTOR signaling involving oligodendroglial cell differentiation-related signaling molecules [27,28] could be improved, differentiation might be promoted. It is possible that modulation of the activities of molecules involved in mTOR signaling or lysosome-related signaling molecules by chemicals, such as ibuprofen or therapeutic RNAs harboring the siRNA backbone could provide therapeutic strategies that work against HLD7.

In the developing brain, HLD7-associated mutations of POLR3A proteins appear to be linked to their decreased transcription abilities for rRNAs and tRNAs and/or downregulation of their expression, leading to insufficient transcription and in turn insufficient translation of many target molecules, especially for myelin-related molecules [9,10]. Similarly, mutations of the gene encoding POLR3B are associated with HLD8 [10,11]. It is likely that HLD8-associated mutations of POLR3B proteins, another component forming the catalytic core of RNA polymerase III, appear to be linked to their decreased transcription abilities for rRNAs and tRNAs or downregulation of their expression. Although RNA polymerase III is needed for every basic cellular function, it remains unclear why abnormal activities of RNA polymerase III are critically associated with oligodendrocyte development, including differentiation and myelination. One of the reasons for this may be that oligodendrocytes result in undergoing morphological changes that generate approximately 10-layered myelin sheaths [5,6,7,8]. It is thought that dynamic morphological changes are usually accomplished with RNA production and, in turn, protein synthesis. Another reason may be that oligodendrocyte RNA polymerase III participates in synthesizing small RNA(s), such as oligodendrocyte-specific miRNAs [37,38]. In either case, it is noteworthy that dynamic morphological changes in oligodendrocytes are very different from those in other cells, possibly utilizing many RNAs.

Here, we have shown that HLD7-associated R140X mutation causes POLR3A proteins to be localized in lysosomes and that they decrease lysosome signal-related molecules belonging to mTOR signaling. Decreased activities of mTOR signaling molecules appear to be responsible for oligodendroglial cell differentiation. In contrast, the treatment of their cells with ibuprofen as the mTOR signaling activator reverses the abilities of the cells to differentiate. These results may suggest one of the possible cellular and molecular pathological mechanisms underlying HLD7 and some strategies for its treatment. Additional detailed studies will allow us to understand the mechanism by which HLD7-associated R140X mutant proteins inhibit differentiation and also how inhibited differentiation is linked to hypomyelination. Further studies may also allow the clarification of possible ibuprofen target molecules other than cyclooxygenases. In the future, it would be useful to determine whether the treatment with ibuprofen is specific for HLD7 or whether it has a broad effect on HLDs in cell levels and model mice. As an extension of these studies, it might be possible to develop new therapeutic drug(s) specific for therapeutic target molecule(s).

## Figures and Tables

**Table 1:** Antibodies and inhibitors.

Product Name	Target or Description	Company	Cat. No.	Lot. No.	Final Conc. for Antibody
Anti-eIF4EBP1 (phospho T37) antibody	A phospho-peptide corresponding to residues surrounding theronine 37 of eIF4EBP1	abcam	ab75767	GR88680-14	IF, 1/100
Anti-F-actin antibody	Filamentous actin (F-actin)	abcam	ab205	GR319251-7	IB, 1/80,000
Anti-GFP mAb	Green fluorescent protein (GFP)	MBL	598	“078”	IB, 1/80,000
Anti-Halo Tag pAb	Halo Tag	Promega	G9281	“0000028445”	IB, 1/20,000
HaloTag Oregon Green Ligand	Halo-tag ligand	Promega	G2802	“0000391849”	IF, 1/1000
Anti-KDEL mAb	KDEL-containing peptide of the endoplasmic reticulum (ER)-resident glucose-regulated protein (GRP78)	MBL	M181-3	“004”	IF, 1/500
Anti-PLP1 antibody	Myelin proteolipid protein 1 (PLP1)	Atlas Antibodies	HPA004128	B115828	IB, 1/500
Anti-RPS6 (phospho S240 + S244) antibody	Synthetic peptide within human S6 protein to the C-terminus (phospho S240 + S244)	abcam	ab215214	GR3205097-3	IF, 1/100
CNPase (D83E10) XP Rabbit mAb	2’, 3’-cyclic nucleotide 3’-phospho-diesterase (CNPase)	Cell Signaling Technology	#5664	1	IB, 1/500
Anti-LAMP-1 antibody (H4A3)	Lysosomal-associated membrane protein1 (LAMP1)	Santa Cruz Biotechnology	sc-20011	J0919	IF, 1/200
Purified Mouse Anti-GM130 antibody	Golgi matrix protein of 130 kDa (GM130)	BD Biosciences	610823	8352796	IF, 1/500
Anti-Sox-10 antibody (A-2)	SRY-related HMG-box 10 (Sox10)	Santa Cruz Biotechnology	sc-365692	J0720	IB, 1/500
Anti-LC3 protein	Microtubule-associated protein 1 light chain 3 (LC3)	MBL	M152-3	“057”	IF, 1/500
Anti-mTOR antibody (A30)	Mechanistic target of rapamycin (mTOR [kinase ])	Santa Cruz Biotechnology	sc-517464	H2621	IB, 1/100
Alexa Fluor 488 goat anti-mouse IgG (H+L)	Mouse IgG (H+L) conjugated with Alexa Fluor 488	Thermo Fisher Scientific	A-11001	774904	IF, 1/500
Alexa Fluor 488 goat anti-rabbit IgG (H+L)	Rabbit IgG (H+L) conjugated with Alexa Fluor 488	Thermo Fisher Scientific	A-11008	751094	IF, 1/500
Alexa Fluor 594 goat anti-mouse IgG (H+L)	Mouse IgG (H+L) conjugated with Alexa Fluor 594	Thermo Fisher Scientific	A-11005	2043369	IF, 1/500
Alexa Fluor 594 goat anti-rabbit IgG (H+L)	Rabbit IgG (H+L) conjugated with Alexa Fluor 594	Thermo Fisher Scientific	A-11012	2018240	IF, 1/500
Anti-IgG (H+L chain) (Mouse) pAb-HRP	Mouse IgG F(ab’) conjugated with horseradish peroxidase	MBL	330	366	IB, 1/5000
Anti-IgG (H+L chain) (Mouse) pAb-HRP	Mouse IgG F(ab’) conjugated with horseradish peroxidase	MBL	330	366	IB, 1/5000
AH6809	Prostaglandin receptor antagonist	Med Chem Express	HY-10418	27839	

## Data Availability

Not applicable.

## Supplementary Materials

The following are available online at https://www.mdpi.com/article/10.3390/neurolint14010002/s1, Figure S1: Expression of wild-type POLR3A proteins does not generate LC3-positive vesicles in cells, Figure S2: Expression of the POLR3A mutant proteins results in generating LC3-positive vesicles in cells, and Figure S3: Full gel scan images.
