# Supplementary material for: Hypomyelinating Leukodystrophy 7 (HLD7)-Associated Mutation of POLR3A Is Related to Defective Oligodendroglial Cell Differentiation, Which Is Ameliorated by Ibuprofen
Source: Neurol Int. 2021 Dec 22;14(1):11–33. doi: 10.3390/neurolint14010002 (PMC8788570; doi:10.3390/neurolint14010002)
Supplement: Supplementary file 1 [file neurolint-14-00002-s001.zip › neurolint-1478260-supplementary.pdf]

Supplemental figure legends

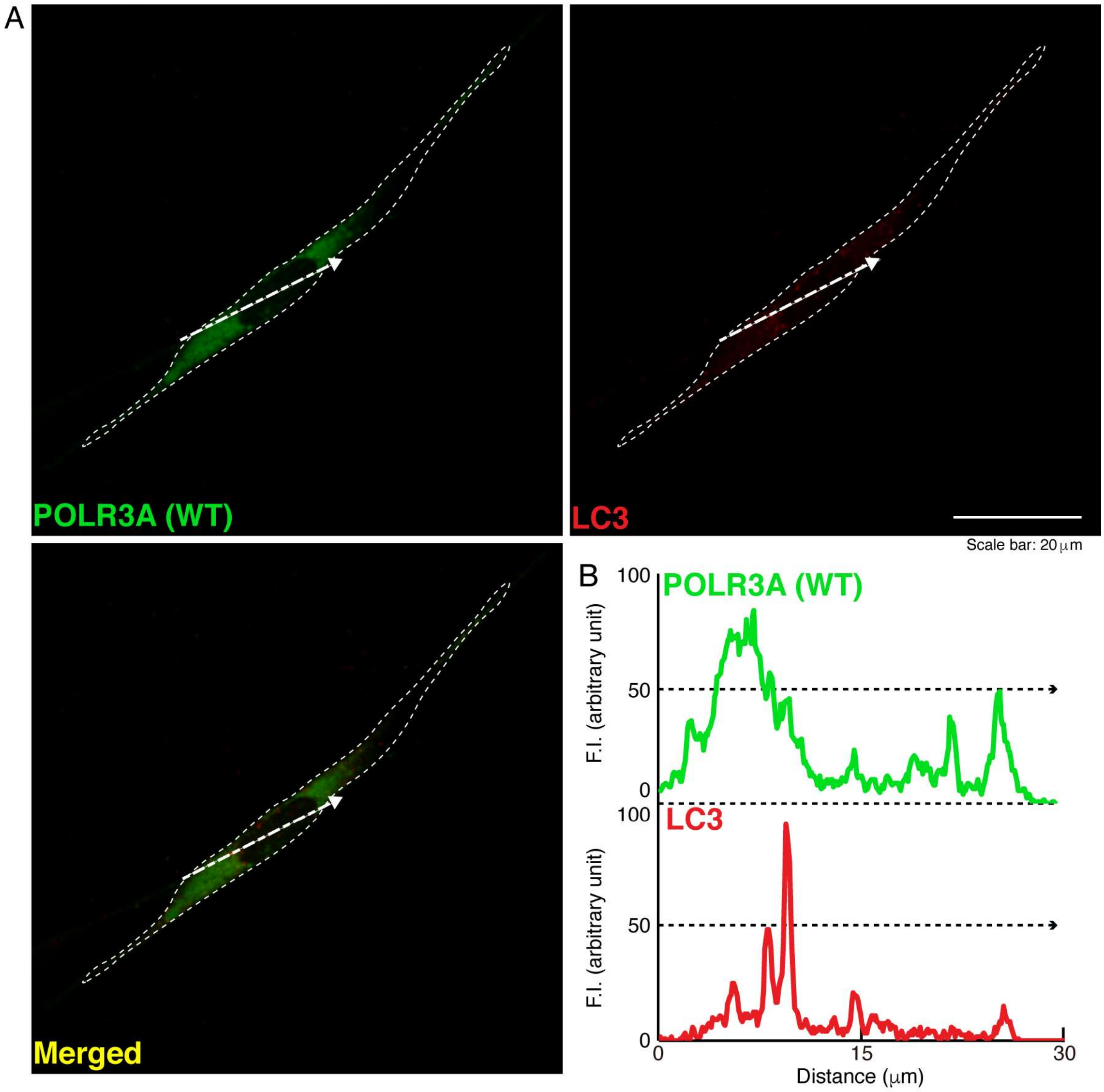

Figure S1. Expression of wild-type POLR3A proteins does not generate LC3-positive vesicles in cells. (A, B) FBD-102b cells were transfected with the plasmid encoding wild-type (WT) POLR3A and stained with transfected proteins (green) and an antibody against the LC3 antigen (red). Scan plots were performed along the white dotted lines in the direction of the arrows in the color images (green and red as well as merged images). Graphs showing the fluorescence intensities (arbitrary units) along the white dotted lines in the direction of the arrows can be seen in the right bottom panels (black dotted lines).

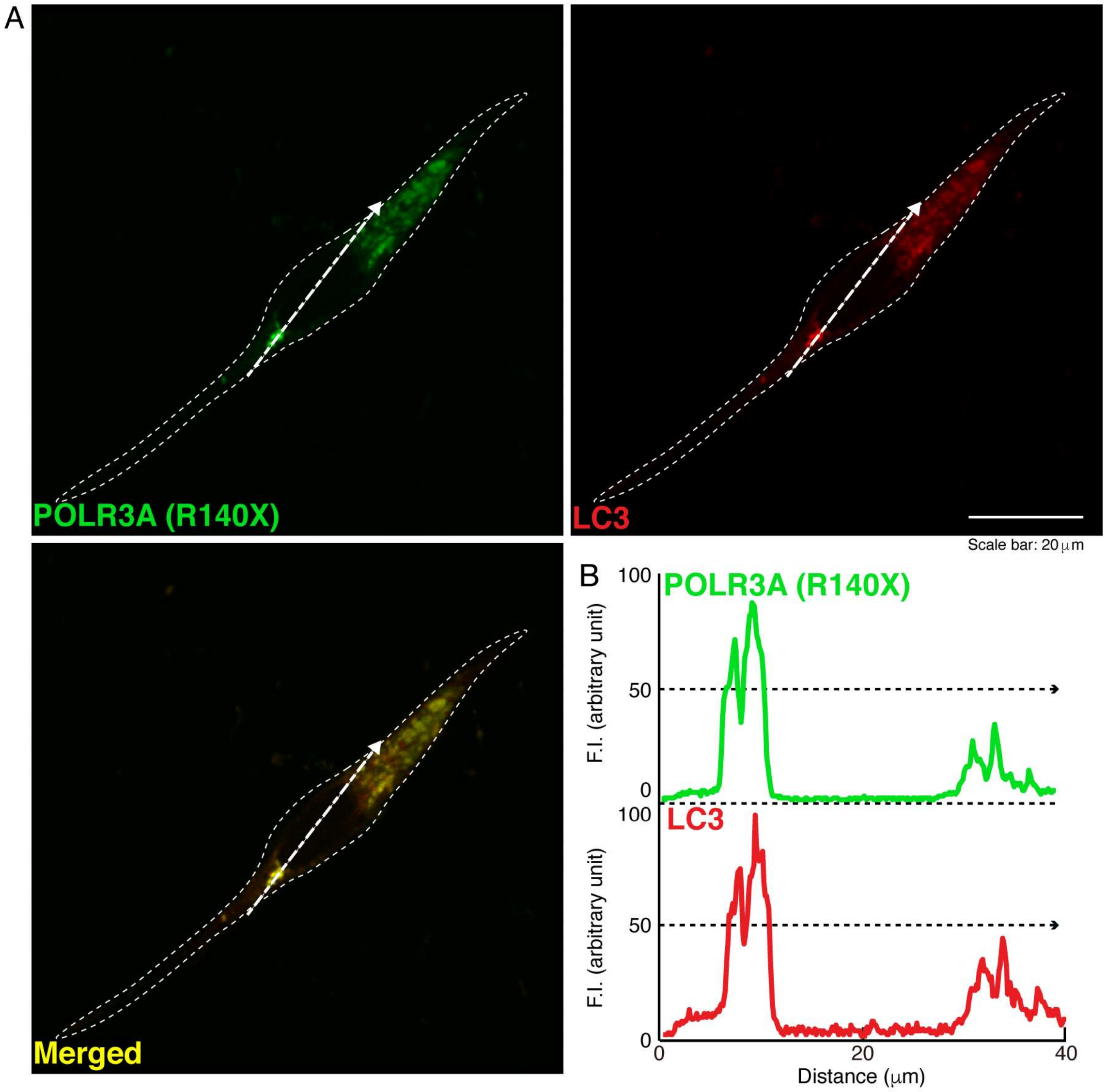

Figure S2. Expression of the POLR3A mutant proteins results in generating LC3-positive vesicles in cells. (A, B) FBD-102b cells were transfected with the plasmid encoding the POLR3A R140X construct (green) and stained using an antibody against the LC3 antigen (red). Scan plots were performed along the white dotted lines in the direction of the arrows in the color images (green and red as well as merged images). Graphs showing the fluorescence intensities (arbitrary units) along the white dotted lines in the direction of the arrows can be seen in the right bottom panels (black dotted).

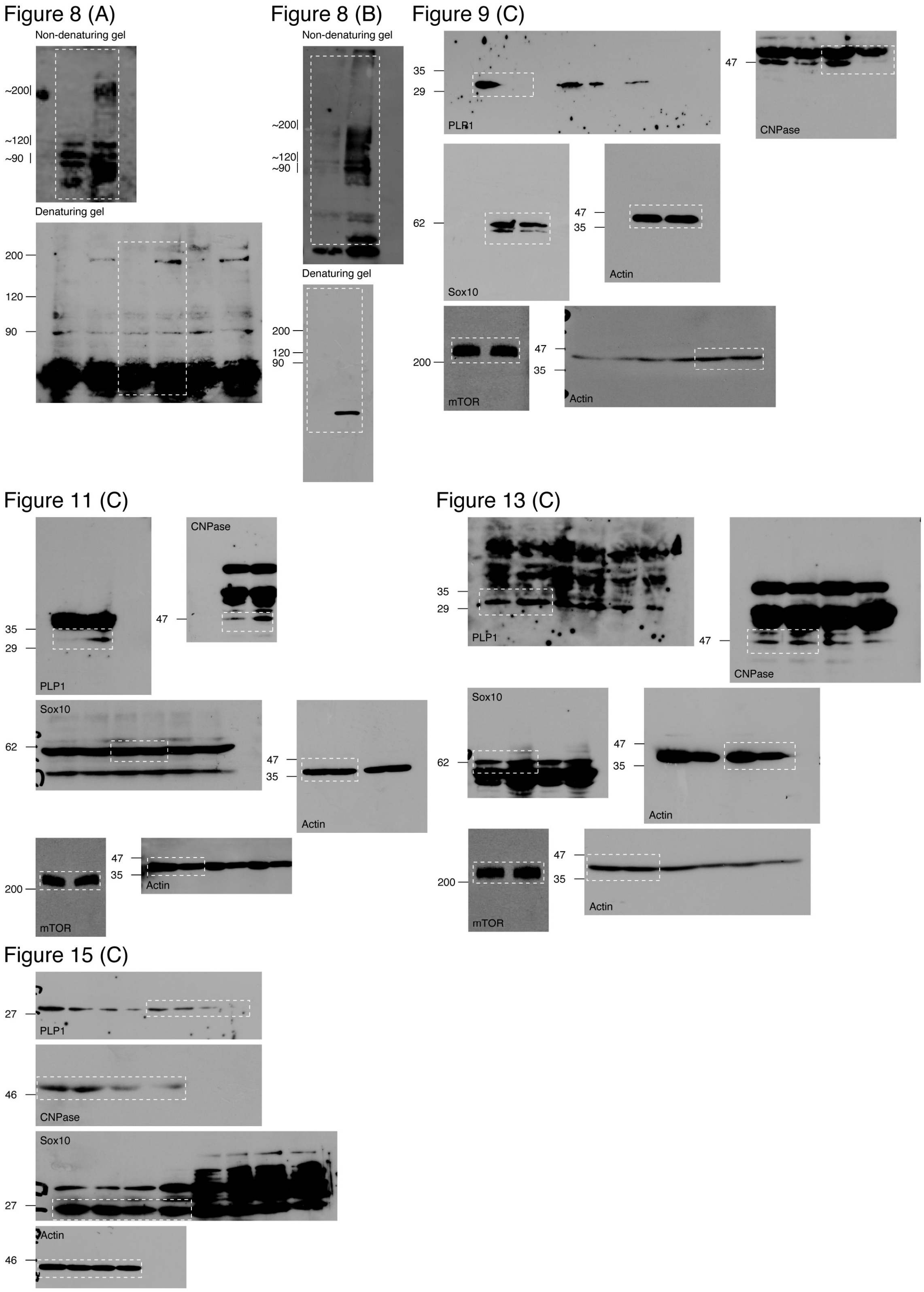

Figure S3. Full gel scan images.
